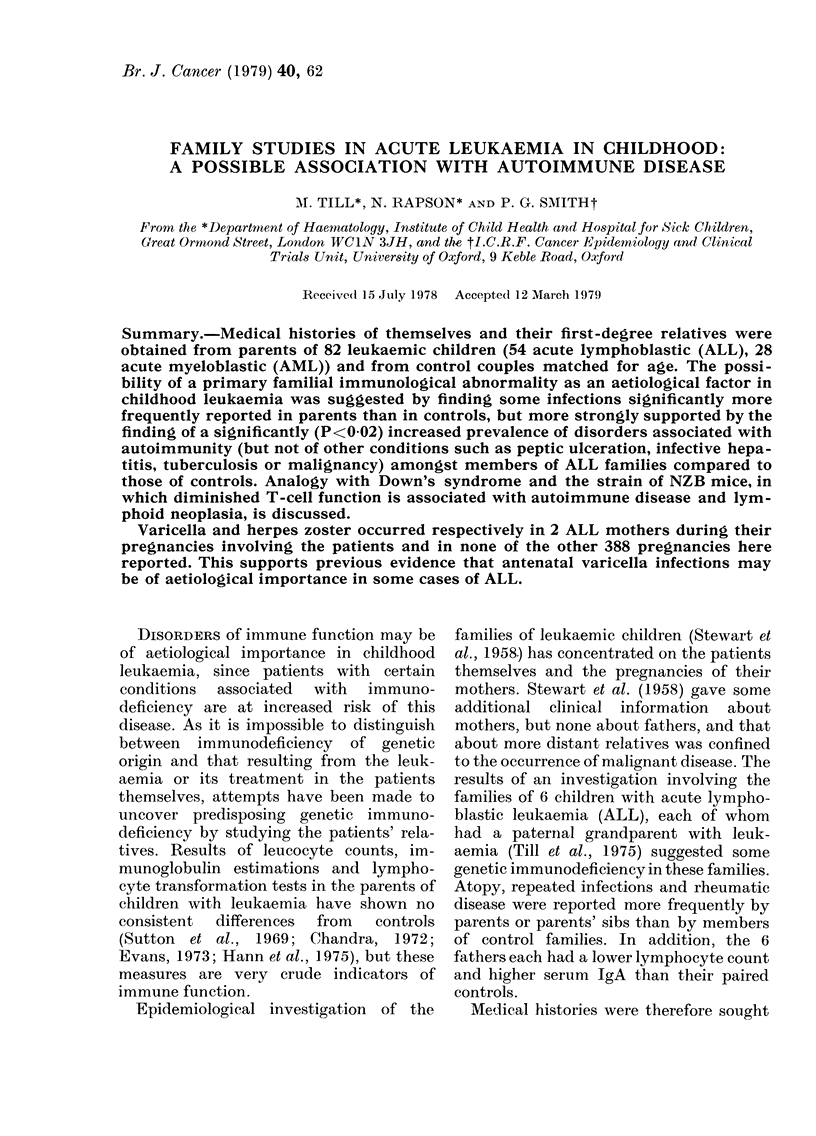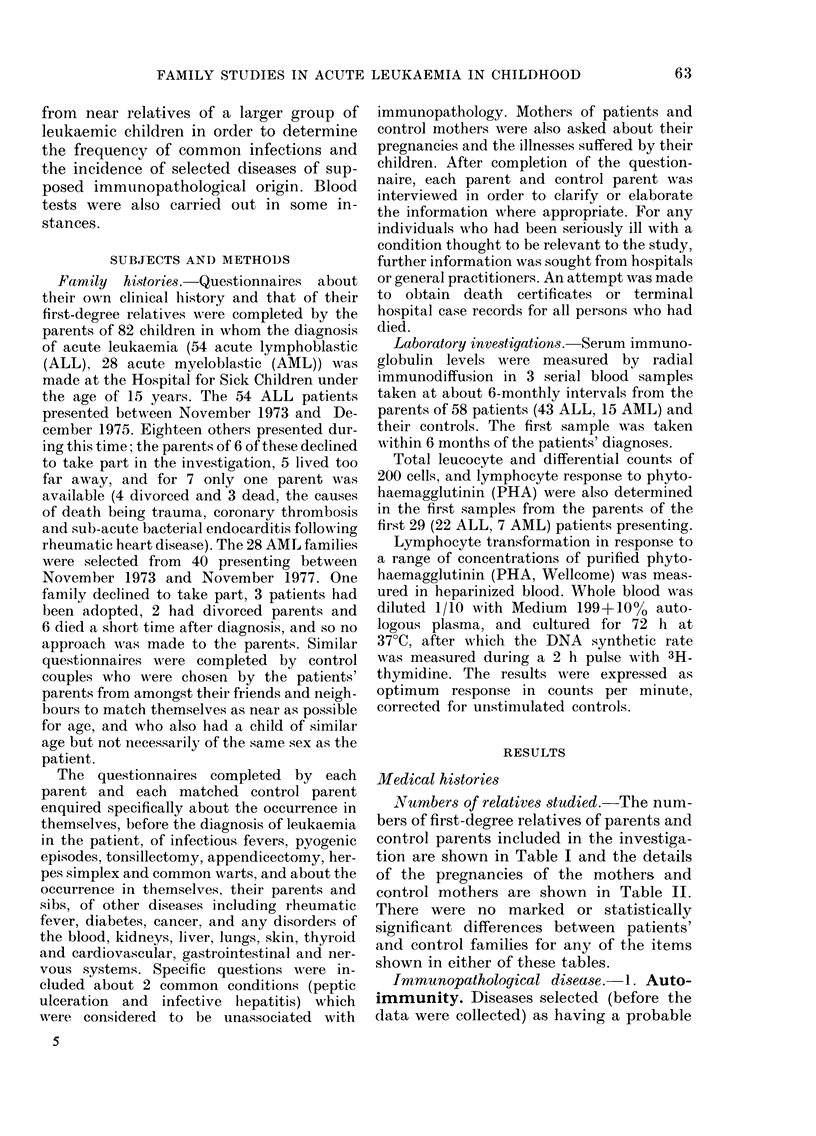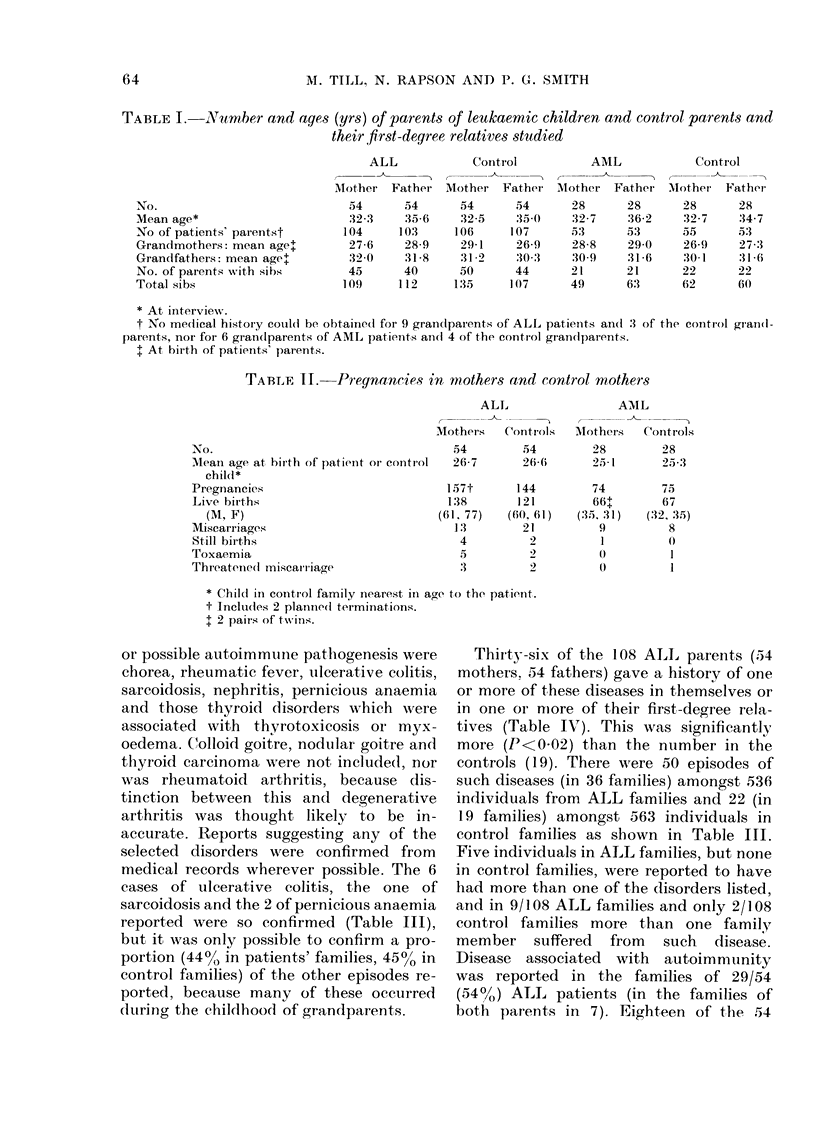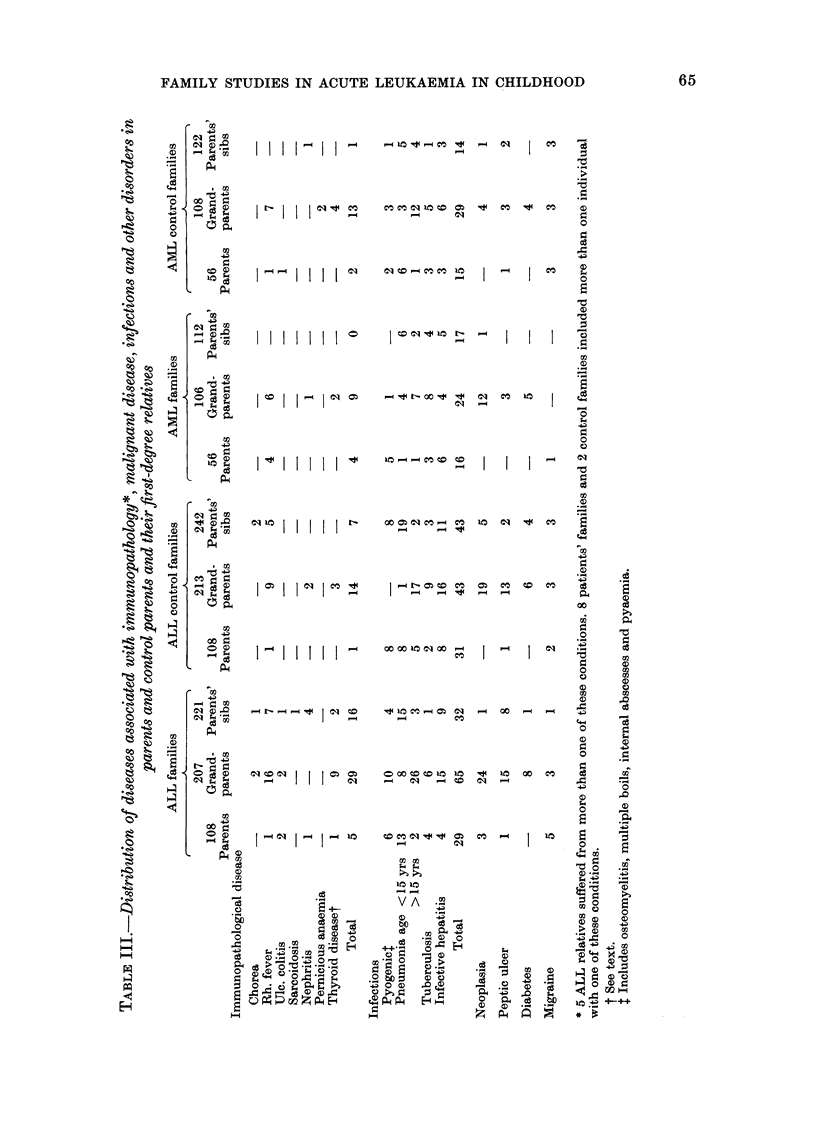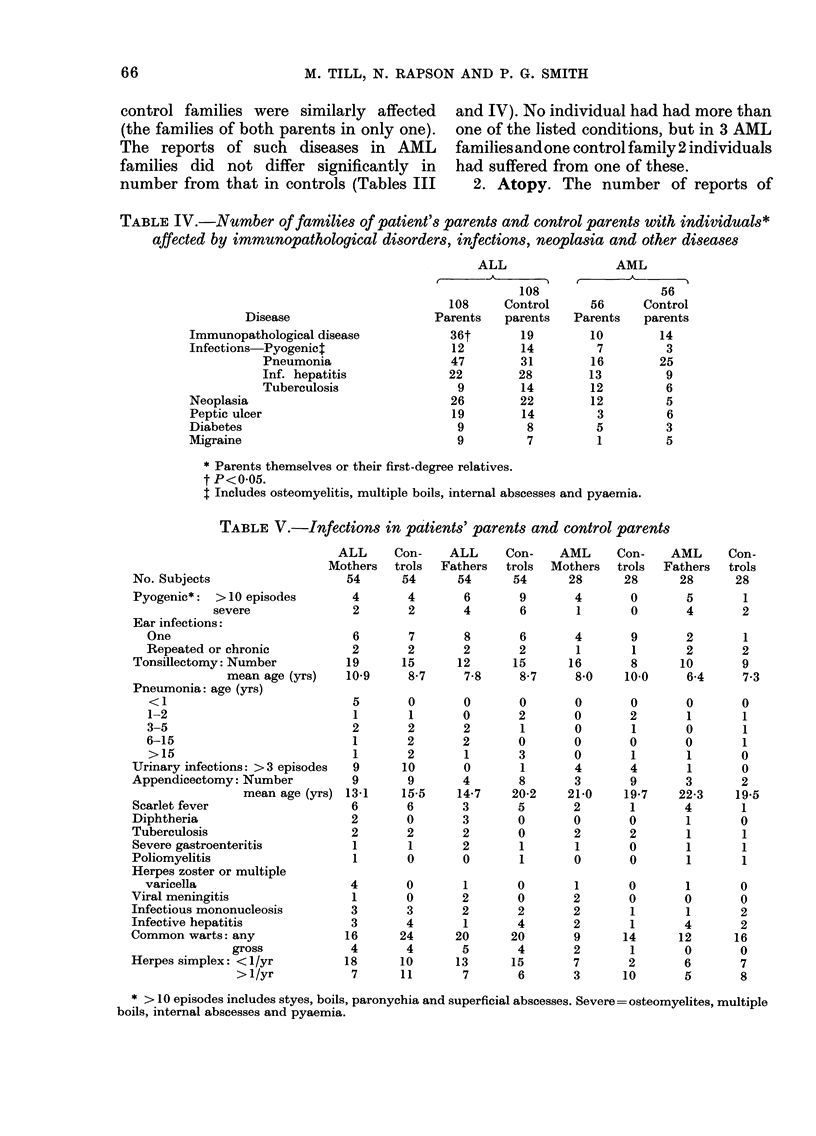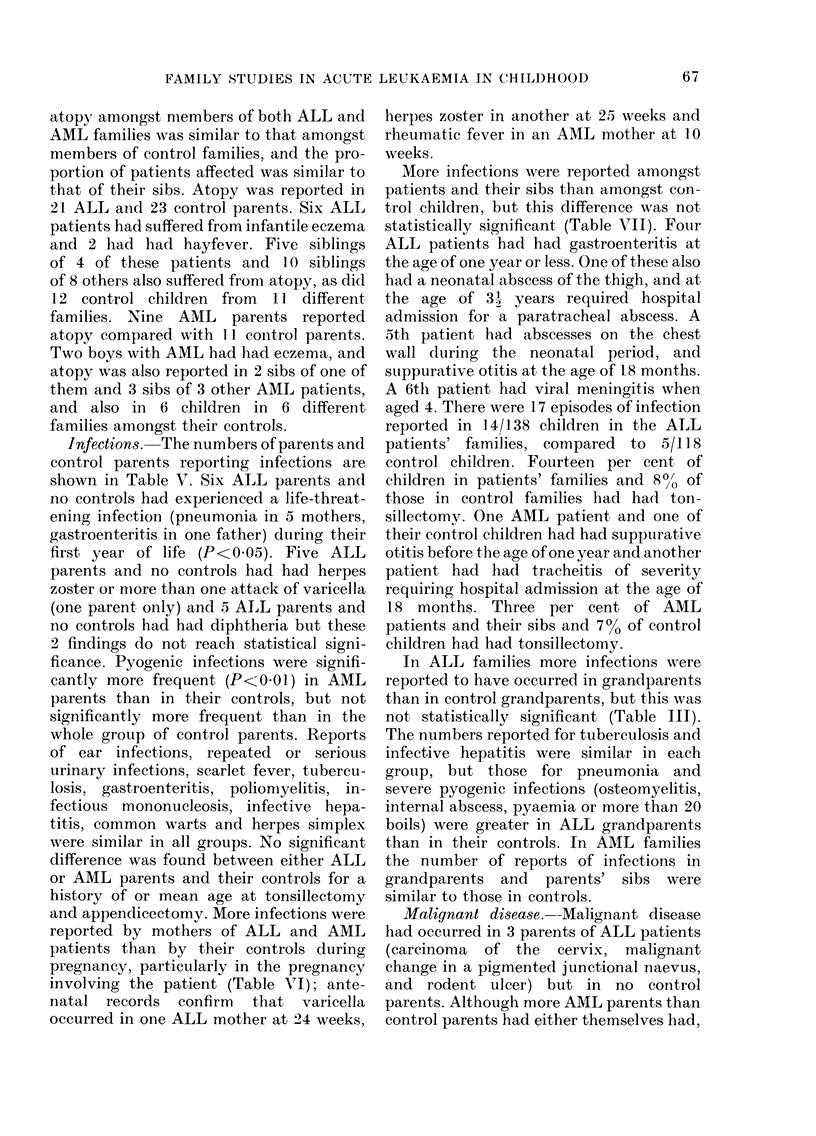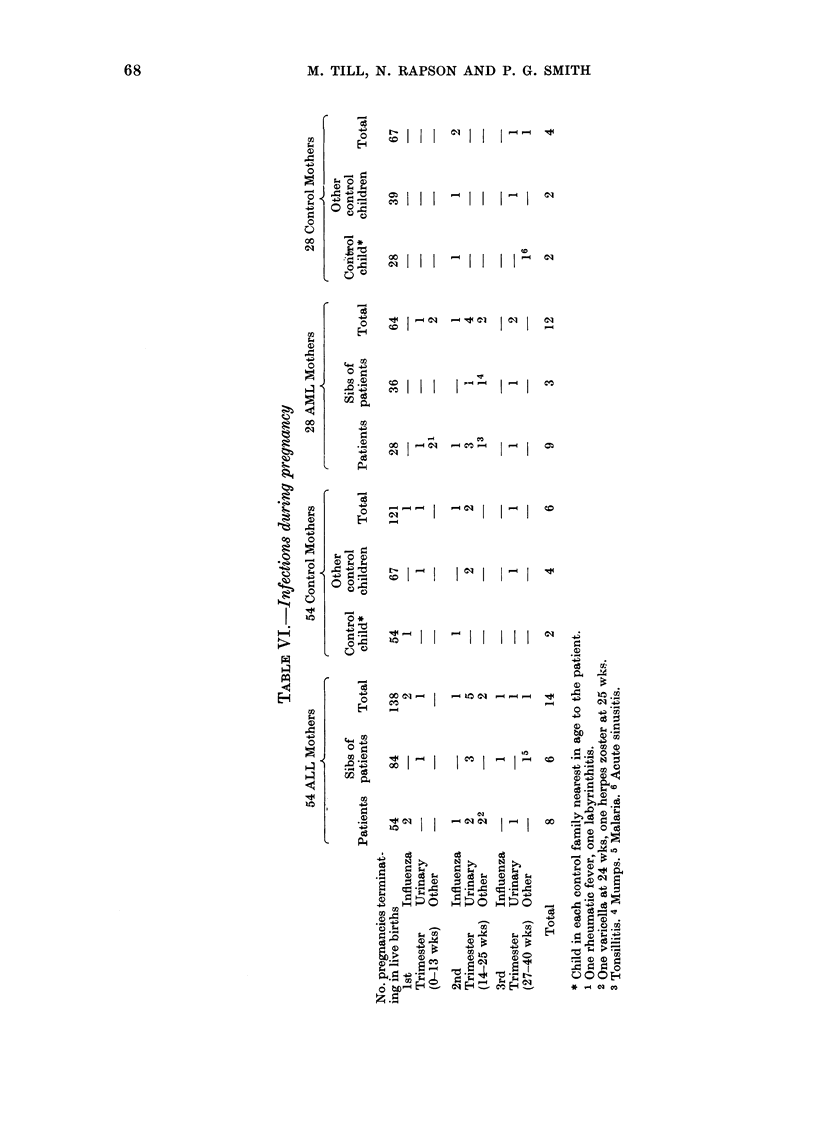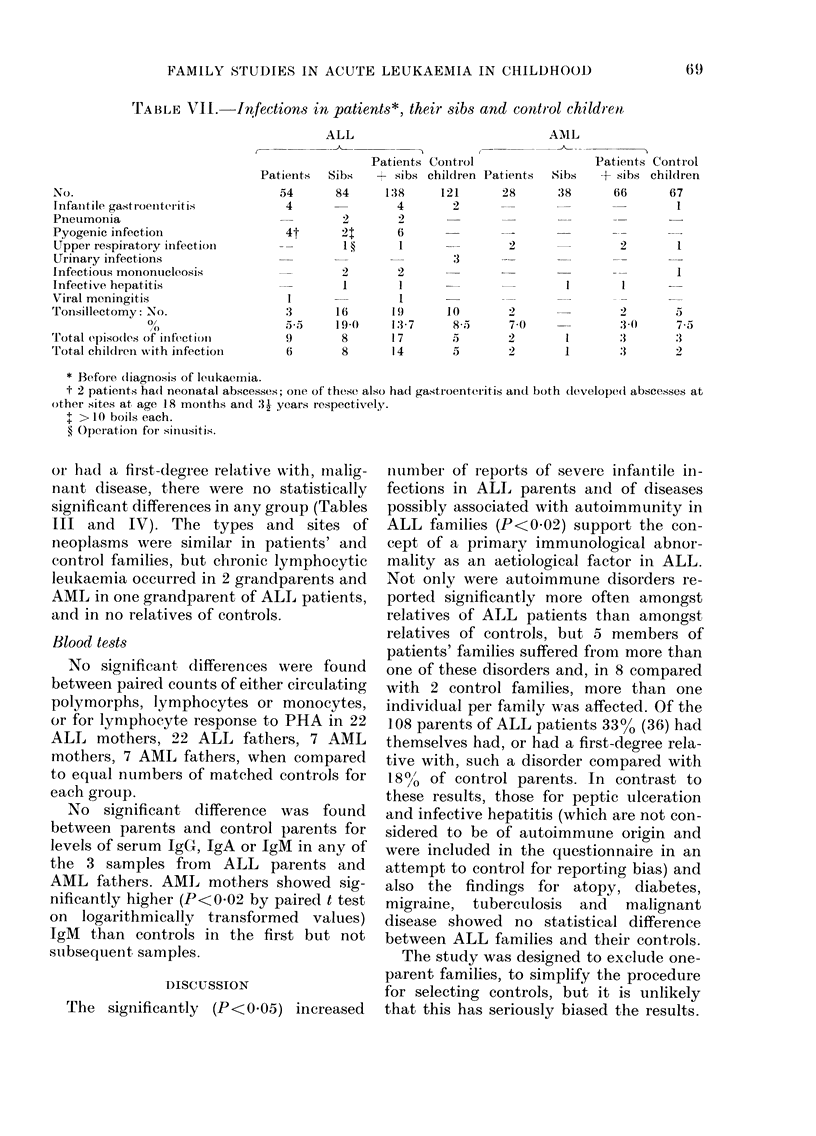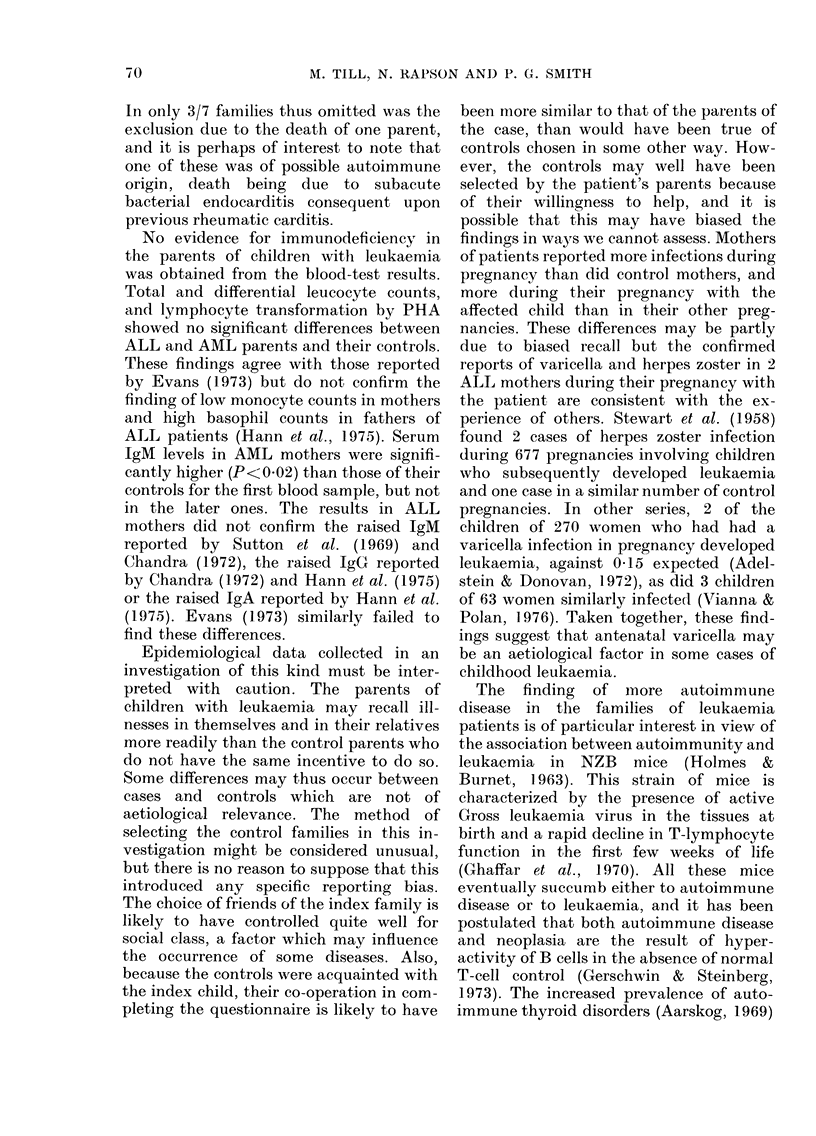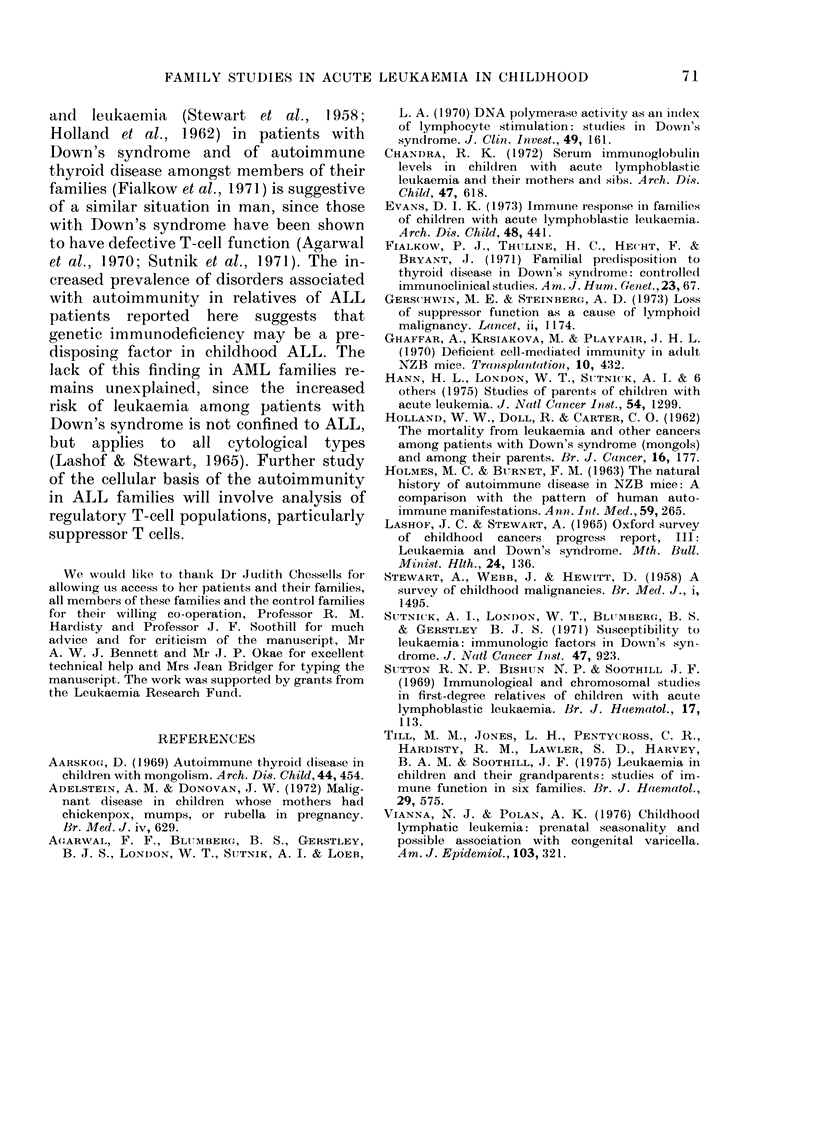# Family studies in acute leukaemia in childhood: a possible association with autoimmune disease.

**DOI:** 10.1038/bjc.1979.141

**Published:** 1979-07

**Authors:** M. Till, N. Rapson, P. G. Smith

## Abstract

Medical histories of themselves and their first-degree relatives were obtained from parents of 82 leukaemic children (54 acute lymphoblastic (ALL), 28 acute myeloblastic (AML)) and from control couples matched for age. The possibility of a primary familial immunological abnormality as an aetiological factor in childhood leukaemia was suggested by binding some infections significantly more frequently reported in parents than in controls, but more strongly supported by the finding of a significantly (P less than 0.02) increased prevalence of disorders associated with autoimmunity (but not of other conditions such as peptic ulceration, infective hepatitis, tuberculosis or malignancy) amongst members of ALL families compared to those of controls. Analogy with Down's syndrome and the strain of NZB mice, in which diminished T-cell function is associated with autoimmune disease and lymphoid neoplasia, is discussed. Varicella and herpes zoster occurred respectively in 2 ALL mothers during their pregnancies involving the patients and in none of the other 388 pregnancies here reported. This supports previous evidence that antenatal varicella infections may be of aetiological importance in some cases of ALL.


*Br. J. Cancer* (1979) **40**, 62

### FAMILY STUDIES IN ACUTE LEUKAEMIA IN CHILDHOOD: A POSSIBLE ASSOCIATION WITH AUTOIMMUNE DISEASE

M. TILL*, N. RAPSON* AND P. G. SMITH†

*From the *Department of Haematology, Institute of Child Health and Hospital for Sick Children, Great Ormond Street, London WC1N 3JH, and the †I.C.R.F. Cancer Epidemiology and Clinical Trials Unit, University of Oxford, 9 Keble Road, Oxford*

Received 15 July 1978 Accepted 12 March 1979

**Summary.—Medical histories of themselves and their first-degree relatives were obtained from parents of 82 leukaemic children (54 acute lymphoblastic (ALL), 28 acute myeloblastic (AML)) and from control couples matched for age. The possibility of a primary familial immunological abnormality as an aetiological factor in childhood leukaemia was suggested by finding some infections significantly more frequently reported in parents than in controls, but more strongly supported by the finding of a significantly ($P<0{\cdot}02$) increased prevalence of disorders associated with autoimmunity (but not of other conditions such as peptic ulceration, infective hepatitis, tuberculosis or malignancy) amongst members of ALL families compared to those of controls. Analogy with Down's syndrome and the strain of NZB mice, in which diminished T-cell function is associated with autoimmune disease and lymphoid neoplasia, is discussed.**

**Varicella and herpes zoster occurred respectively in 2 ALL mothers during their pregnancies involving the patients and in none of the other 388 pregnancies here reported. This supports previous evidence that antenatal varicella infections may be of aetiological importance in some cases of ALL.**

DISORDERS of immune function may be of aetiological importance in childhood leukaemia, since patients with certain conditions associated with immunodeficiency are at increased risk of this disease. As it is impossible to distinguish between immunodeficiency of genetic origin and that resulting from the leukaemia or its treatment in the patients themselves, attempts have been made to uncover predisposing genetic immunodeficiency by studying the patients' relatives. Results of leucocyte counts, immunoglobulin estimations and lymphocyte transformation tests in the parents of children with leukaemia have shown no consistent differences from controls (Sutton *et al.*, 1969; Chandra, 1972; Evans, 1973; Hann *et al.*, 1975), but these measures are very crude indicators of immune function.

Epidemiological investigation of the families of leukaemic children (Stewart *et al.*, 1958) has concentrated on the patients themselves and the pregnancies of their mothers. Stewart *et al.* (1958) gave some additional clinical information about mothers, but none about fathers, and that about more distant relatives was confined to the occurrence of malignant disease. The results of an investigation involving the families of 6 children with acute lymphoblastic leukaemia (ALL), each of whom had a paternal grandparent with leukaemia (Till *et al.*, 1975) suggested some genetic immunodeficiency in these families. Atopy, repeated infections and rheumatic disease were reported more frequently by parents or parents' sibs than by members of control families. In addition, the 6 fathers each had a lower lymphocyte count and higher serum IgA than their paired controls.

Medical histories were therefore sought from near relatives of a larger group of leukaemic children in order to determine the frequency of common infections and the incidence of selected diseases of supposed immunopathological origin. Blood tests were also carried out in some instances.

### SUBJECTS AND METHODS

*Family histories.*—Questionnaires about their own clinical history and that of their first-degree relatives were completed by the parents of 82 children in whom the diagnosis of acute leukaemia (54 acute lymphoblastic (ALL), 28 acute myeloblastic (AML)) was made at the Hospital for Sick Children under the age of 15 years. The 54 ALL patients presented between November 1973 and December 1975. Eighteen others presented during this time; the parents of 6 of these declined to take part in the investigation, 5 lived too far away, and for 7 only one parent was available (4 divorced and 3 dead, the causes of death being trauma, coronary thrombosis and sub-acute bacterial endocarditis following rheumatic heart disease). The 28 AML families were selected from 40 presenting between November 1973 and November 1977. One family declined to take part, 3 patients had been adopted, 2 had divorced parents and 6 died a short time after diagnosis, and so no approach was made to the parents. Similar questionnaires were completed by control couples who were chosen by the patients' parents from amongst their friends and neighbours to match themselves as near as possible for age, and who also had a child of similar age but not necessarily of the same sex as the patient.

The questionnaires completed by each parent and each matched control parent enquired specifically about the occurrence in themselves, before the diagnosis of leukaemia in the patient, of infectious fevers, pyogenic episodes, tonsillectomy, appendicectomy, herpes simplex and common warts, and about the occurrence in themselves, their parents and sibs, of other diseases including rheumatic fever, diabetes, cancer, and any disorders of the blood, kidneys, liver, lungs, skin, thyroid and cardiovascular, gastrointestinal and nervous systems. Specific questions were included about 2 common conditions (peptic ulceration and infective hepatitis) which were considered to be unassociated with immunopathology. Mothers of patients and control mothers were also asked about their pregnancies and the illnesses suffered by their children. After completion of the questionnaire, each parent and control parent was interviewed in order to clarify or elaborate the information where appropriate. For any individuals who had been seriously ill with a condition thought to be relevant to the study, further information was sought from hospitals or general practitioners. An attempt was made to obtain death certificates or terminal hospital case records for all persons who had died.

*Laboratory investigations.*—Serum immunoglobulin levels were measured by radial immunodiffusion in 3 serial blood samples taken at about 6-monthly intervals from the parents of 58 patients (43 ALL, 15 AML) and their controls. The first sample was taken within 6 months of the patients' diagnoses.

Total leucocyte and differential counts of 200 cells, and lymphocyte response to phytohaemagglutinin (PHA) were also determined in the first samples from the parents of the first 29 (22 ALL, 7 AML) patients presenting.

Lymphocyte transformation in response to a range of concentrations of purified phytohaemagglutinin (PHA, Wellcome) was measured in heparinized blood. Whole blood was diluted 1/10 with Medium 199+10% autologous plasma, and cultured for 72 h at 37°C, after which the DNA synthetic rate was measured during a 2 h pulse with $^{3}$H-thymidine. The results were expressed as optimum response in counts per minute, corrected for unstimulated controls.

### RESULTS

### *Medical histories*

*Numbers of relatives studied.*—The numbers of first-degree relatives of parents and control parents included in the investigation are shown in Table I and the details of the pregnancies of the mothers and control mothers are shown in Table II. There were no marked or statistically significant differences between patients' and control families for any of the items shown in either of these tables.

*Immunopathological disease.*—1. **Autoimmunity.** Diseases selected (before the data were collected) as having a probable

TABLE I.—*Number and ages (yrs) of parents of leukaemic children and control parents and their first-degree relatives studied*

| | ALL | | Control | | AML | | Control | |
|---|---|---|---|---|---|---|---|---|
| | Mother | Father | Mother | Father | Mother | Father | Mother | Father |
| No. | 54 | 54 | 54 | 54 | 28 | 28 | 28 | 28 |
| Mean age* | 32·3 | 35·6 | 32·5 | 35·0 | 32·7 | 36·2 | 32·7 | 34·7 |
| No of patients' parents† | 104 | 103 | 106 | 107 | 53 | 53 | 55 | 53 |
| Grandmothers: mean age‡ | 27·6 | 28·9 | 29·1 | 26·9 | 28·8 | 29·0 | 26·9 | 27·3 |
| Grandfathers: mean age‡ | 32·0 | 31·8 | 31·2 | 30·3 | 30·9 | 31·6 | 30·1 | 31·6 |
| No. of parents with sibs | 45 | 40 | 50 | 44 | 21 | 21 | 22 | 22 |
| Total sibs | 109 | 112 | 135 | 107 | 49 | 63 | 62 | 60 |

* At interview.
† No medical history could be obtained for 9 grandparents of ALL patients and 3 of the control grandparents, nor for 6 grandparents of AML patients and 4 of the control grandparents.
‡ At birth of patients' parents.

TABLE II.—*Pregnancies in mothers and control mothers*

| | ALL | | AML | |
|---|---|---|---|---|
| | Mothers | Controls | Mothers | Controls |
| No. | 54 | 54 | 28 | 28 |
| Mean age at birth of patient or control child* | 26·7 | 26·6 | 25·1 | 25·3 |
| Pregnancies | 157† | 144 | 74 | 75 |
| Live births | 138 | 121 | 66‡ | 67 |
| (M, F) | (61, 77) | (60, 61) | (35, 31) | (32, 35) |
| Miscarriages | 13 | 21 | 9 | 8 |
| Still births | 4 | 2 | 1 | 0 |
| Toxaemia | 5 | 2 | 0 | 1 |
| Threatened miscarriage | 3 | 2 | 0 | 1 |

* Child in control family nearest in age to the patient.

† Includes 2 planned terminations.

‡ 2 pairs of twins.

or possible autoimmune pathogenesis were chorea, rheumatic fever, ulcerative colitis, sarcoidosis, nephritis, pernicious anaemia and those thyroid disorders which were associated with thyrotoxicosis or myxoedema. Colloid goitre, nodular goitre and thyroid carcinoma were not included, nor was rheumatoid arthritis, because distinction between this and degenerative arthritis was thought likely to be inaccurate. Reports suggesting any of the selected disorders were confirmed from medical records wherever possible. The 6 cases of ulcerative colitis, the one of sarcoidosis and the 2 of pernicious anaemia reported were so confirmed (Table III), but it was only possible to confirm a proportion (44% in patients' families, 45% in control families) of the other episodes reported, because many of these occurred during the childhood of grandparents.

Thirty-six of the 108 ALL parents (54 mothers, 54 fathers) gave a history of one or more of these diseases in themselves or in one or more of their first-degree relatives (Table IV). This was significantly more ($P<0{\cdot}02$) than the number in the controls (19). There were 50 episodes of such diseases (in 36 families) amongst 536 individuals from ALL families and 22 (in 19 families) amongst 563 individuals in control families as shown in Table III. Five individuals in ALL families, but none in control families, were reported to have had more than one of the disorders listed, and in 9/108 ALL families and only 2/108 control families more than one family member suffered from such disease. Disease associated with autoimmunity was reported in the families of 29/54 (54%) ALL patients (in the families of both parents in 7). Eighteen of the 54

TABLE III.—*Distribution of diseases associated with immunopathology*, malignant disease, infections and other disorders in parents and control parents and their first-degree relatives*

| | ALL families | | | ALL control families | | | AML families | | | AML control families | | |
|---|---|---|---|---|---|---|---|---|---|---|---|---|
| | 108 Parents | 207 Grand-parents | 221 Parents' sibs | 108 Parents | 213 Grand-parents | 242 Parents' sibs | 56 Parents | 106 Grand-parents | 112 Parents' sibs | 56 Parents | 108 Grand-parents | 122 Parents' sibs |
| Immunopathological disease | | | | | | | | | | | | |
| Chorea | — | 2 | 1 | — | — | 2 | — | — | — | — | — | — |
| Rh. fever | 1 | 16 | 7 | 1 | 9 | 5 | 4 | 6 | — | 1 | 7 | — |
| Ulc. colitis | 2 | 2 | 1 | — | — | — | — | — | — | 1 | — | — |
| Sarcoidosis | — | — | 1 | — | — | — | — | — | — | — | — | — |
| Nephritis | 1 | — | 4 | — | 2 | — | — | 1 | — | — | — | 1 |
| Pernicious anaemia | — | — | — | — | — | — | — | — | — | — | 2 | — |
| Thyroid disease† | 1 | 9 | 2 | — | 3 | — | — | 2 | — | — | 4 | — |
| Total | 5 | 29 | 16 | 1 | 14 | 7 | 4 | 9 | 0 | 2 | 13 | 1 |
| Infections | | | | | | | | | | | | |
| Pyogenic‡ | 6 | 10 | 4 | 8 | — | 8 | 5 | 1 | — | 2 | 3 | 1 |
| Pneumonia age <15 yrs | 13 | 8 | 15 | 8 | 1 | 19 | 1 | 4 | 6 | 6 | 3 | 5 |
| >15 yrs | 2 | 26 | 3 | 5 | 17 | 2 | 1 | 7 | 2 | 1 | 12 | 4 |
| Tuberculosis | 4 | 6 | 1 | 2 | 9 | 3 | 3 | 8 | 4 | 3 | 5 | 1 |
| Infective hepatitis | 4 | 15 | 9 | 8 | 16 | 11 | 6 | 4 | 5 | 3 | 6 | 3 |
| Total | 29 | 65 | 32 | 31 | 43 | 43 | 16 | 24 | 17 | 15 | 29 | 14 |
| Neoplasia | 3 | 24 | 1 | — | 19 | 5 | — | 12 | 1 | — | 4 | 1 |
| Peptic ulcer | 1 | 15 | 8 | 1 | 13 | 2 | — | 3 | — | 1 | 3 | 2 |
| Diabetes | — | 8 | 1 | — | 6 | 4 | — | 5 | — | — | 4 | — |
| Migraine | 5 | 3 | 1 | 2 | 3 | 3 | 1 | — | — | 3 | 3 | 3 |

* 5 ALL relatives suffered from more than one of these conditions. 8 patients' families and 2 control families included more than one individual with one of these conditions.

† See text.

‡ Includes osteomyelitis, multiple boils, internal abscesses and pyaemia.

control families were similarly affected (the families of both parents in only one). The reports of such diseases in AML families did not differ significantly in number from that in controls (Tables III and IV). No individual had had more than one of the listed conditions, but in 3 AML families and one control family 2 individuals had suffered from one of these.

2. **Atopy**. The number of reports of

TABLE IV.—*Number of families of patient's parents and control parents with individuals* affected by immunopathological disorders, infections, neoplasia and other diseases*

| | ALL | | AML | |
|---|---|---|---|---|
| Disease | 108 Parents | 108 Control parents | 56 Parents | 56 Control parents |
| Immunopathological disease | 36† | 19 | 10 | 14 |
| Infections—Pyogenic‡ | 12 | 14 | 7 | 3 |
| Pneumonia | 47 | 31 | 16 | 25 |
| Inf. hepatitis | 22 | 28 | 13 | 9 |
| Tuberculosis | 9 | 14 | 12 | 6 |
| Neoplasia | 26 | 22 | 12 | 5 |
| Peptic ulcer | 19 | 14 | 3 | 6 |
| Diabetes | 9 | 8 | 5 | 3 |
| Migraine | 9 | 7 | 1 | 5 |

* Parents themselves or their first-degree relatives.
† $P<0{\cdot}05$.
‡ Includes osteomyelitis, multiple boils, internal abscesses and pyaemia.

TABLE V.—*Infections in patients' parents and control parents*

| | ALL Mothers | Controls | ALL Fathers | Controls | AML Mothers | Controls | AML Fathers | Controls |
|---|---|---|---|---|---|---|---|---|
| No. Subjects | 54 | 54 | 54 | 54 | 28 | 28 | 28 | 28 |
| Pyogenic*: >10 episodes | 4 | 4 | 6 | 9 | 4 | 0 | 5 | 1 |
| severe | 2 | 2 | 4 | 6 | 1 | 0 | 4 | 2 |
| Ear infections: | | | | | | | | |
| One | 6 | 7 | 8 | 6 | 4 | 9 | 2 | 1 |
| Repeated or chronic | 2 | 2 | 2 | 2 | 1 | 1 | 2 | 2 |
| Tonsillectomy: Number | 19 | 15 | 12 | 15 | 16 | 8 | 10 | 9 |
| mean age (yrs) | 10·9 | 8·7 | 7·8 | 8·7 | 8·0 | 10·0 | 6·4 | 7·3 |
| Pneumonia: age (yrs) | | | | | | | | |
| <1 | 5 | 0 | 0 | 0 | 0 | 0 | 0 | 0 |
| 1–2 | 1 | 1 | 0 | 2 | 0 | 2 | 1 | 1 |
| 3–5 | 2 | 2 | 2 | 1 | 0 | 1 | 0 | 1 |
| 6–15 | 1 | 2 | 2 | 0 | 0 | 0 | 0 | 1 |
| >15 | 1 | 2 | 1 | 3 | 0 | 1 | 1 | 0 |
| Urinary infections: >3 episodes | 9 | 10 | 0 | 1 | 4 | 4 | 1 | 0 |
| Appendicectomy: Number | 9 | 9 | 4 | 8 | 3 | 9 | 3 | 2 |
| mean age (yrs) | 13·1 | 15·5 | 14·7 | 20·2 | 21·0 | 19·7 | 22·3 | 19·5 |
| Scarlet fever | 6 | 6 | 3 | 5 | 2 | 1 | 4 | 1 |
| Diphtheria | 2 | 0 | 3 | 0 | 0 | 0 | 1 | 0 |
| Tuberculosis | 2 | 2 | 2 | 0 | 2 | 2 | 1 | 1 |
| Severe gastroenteritis | 1 | 1 | 2 | 1 | 1 | 0 | 1 | 1 |
| Poliomyelitis | 1 | 0 | 0 | 1 | 0 | 0 | 1 | 1 |
| Herpes zoster or multiple varicella | 4 | 0 | 1 | 0 | 1 | 0 | 1 | 0 |
| Viral meningitis | 1 | 0 | 2 | 0 | 2 | 0 | 0 | 0 |
| Infectious mononucleosis | 3 | 3 | 2 | 2 | 2 | 1 | 1 | 2 |
| Infective hepatitis | 3 | 4 | 1 | 4 | 2 | 1 | 4 | 2 |
| Common warts: any | 16 | 24 | 20 | 20 | 9 | 14 | 12 | 16 |
| gross | 4 | 4 | 5 | 4 | 2 | 1 | 0 | 0 |
| Herpes simplex: <1/yr | 18 | 10 | 13 | 15 | 7 | 2 | 6 | 7 |
| >1/yr | 7 | 11 | 7 | 6 | 3 | 10 | 5 | 8 |

* >10 episodes includes styes, boils, paronychia and superficial abscesses. Severe=osteomyelites, multiple boils, internal abscesses and pyaemia.

atopy amongst members of both ALL and AML families was similar to that amongst members of control families, and the proportion of patients affected was similar to that of their sibs. Atopy was reported in 21 ALL and 23 control parents. Six ALL patients had suffered from infantile eczema and 2 had had hayfever. Five siblings of 4 of these patients and 10 siblings of 8 others also suffered from atopy, as did 12 control children from 11 different families. Nine AML parents reported atopy compared with 11 control parents. Two boys with AML had had eczema, and atopy was also reported in 2 sibs of one of them and 3 sibs of 3 other AML patients, and also in 6 children in 6 different families amongst their controls.

*Infections.*—The numbers of parents and control parents reporting infections are shown in Table V. Six ALL parents and no controls had experienced a life-threatening infection (pneumonia in 5 mothers, gastroenteritis in one father) during their first year of life ($P<0{\cdot}05$). Five ALL parents and no controls had had herpes zoster or more than one attack of varicella (one parent only) and 5 ALL parents and no controls had had diphtheria but these 2 findings do not reach statistical significance. Pyogenic infections were significantly more frequent ($P<0{\cdot}01$) in AML parents than in their controls, but not significantly more frequent than in the whole group of control parents. Reports of ear infections, repeated or serious urinary infections, scarlet fever, tuberculosis, gastroenteritis, poliomyelitis, infectious mononucleosis, infective hepatitis, common warts and herpes simplex were similar in all groups. No significant difference was found between either ALL or AML parents and their controls for a history of or mean age at tonsillectomy and appendicectomy. More infections were reported by mothers of ALL and AML patients than by their controls during pregnancy, particularly in the pregnancy involving the patient (Table VI); antenatal records confirm that varicella occurred in one ALL mother at 24 weeks, herpes zoster in another at 25 weeks and rheumatic fever in an AML mother at 10 weeks.

More infections were reported amongst patients and their sibs than amongst control children, but this difference was not statistically significant (Table VII). Four ALL patients had had gastroenteritis at the age of one year or less. One of these also had a neonatal abscess of the thigh, and at the age of $3\frac{1}{2}$ years required hospital admission for a paratracheal abscess. A 5th patient had abscesses on the chest wall during the neonatal period, and suppurative otitis at the age of 18 months. A 6th patient had viral meningitis when aged 4. There were 17 episodes of infection reported in 14/138 children in the ALL patients' families, compared to 5/118 control children. Fourteen per cent of children in patients' families and 8% of those in control families had had tonsillectomy. One AML patient and one of their control children had had suppurative otitis before the age of one year and another patient had had tracheitis of severity requiring hospital admission at the age of 18 months. Three per cent of AML patients and their sibs and 7% of control children had had tonsillectomy.

In ALL families more infections were reported to have occurred in grandparents than in control grandparents, but this was not statistically significant (Table III). The numbers reported for tuberculosis and infective hepatitis were similar in each group, but those for pneumonia and severe pyogenic infections (osteomyelitis, internal abscess, pyaemia or more than 20 boils) were greater in ALL grandparents than in their controls. In AML families the number of reports of infections in grandparents and parents' sibs were similar to those in controls.

*Malignant disease.*—Malignant disease had occurred in 3 parents of ALL patients (carcinoma of the cervix, malignant change in a pigmented junctional naevus, and rodent ulcer) but in no control parents. Although more AML parents than control parents had either themselves had,

TABLE VI.—*Infections during pregnancy*

| | | 54 ALL Mothers | | | 54 Control Mothers | | | 28 AML Mothers | | | 28 Control Mothers | | |
|---|---|---|---|---|---|---|---|---|---|---|---|---|---|
| | | Patients | Sibs of patients | Total | Control child* | Other control children | Total | Patients | Sibs of patients | Total | Control child* | Other control children | Total |
| No. pregnancies terminating in live births | | 54 | 84 | 138 | 54 | 67 | 121 | 28 | 36 | 64 | 28 | 39 | 67 |
| 1st | Influenza | 2 | — | 2 | 1 | — | 1 | — | — | — | — | — | — |
| Trimester | Urinary | — | 1 | 1 | — | 1 | 1 | 1 | — | 1 | — | — | — |
| (0–13 wks) | Other | — | — | — | — | — | — | 2[1] | — | 2 | — | — | — |
| 2nd | Influenza | 1 | — | 1 | 1 | — | 1 | 1 | — | 1 | 1 | 1 | 2 |
| Trimester | Urinary | 2 | 3 | 5 | — | 2 | 2 | 3 | 1 | 4 | — | — | — |
| (14–25 wks) | Other | 2[2] | — | 2 | — | — | — | 1[3] | 1[4] | 2 | — | — | — |
| 3rd | Influenza | — | 1 | 1 | — | — | — | — | — | — | — | — | — |
| Trimester | Urinary | 1 | — | 1 | — | 1 | 1 | 1 | 1 | 2 | — | 1 | 1 |
| (27–40 wks) | Other | — | 1[5] | 1 | — | — | — | — | — | — | 1[6] | — | 1 |
| Total | | 8 | 6 | 14 | 2 | 4 | 6 | 9 | 3 | 12 | 2 | 2 | 4 |

* Child in each control family nearest in age to the patient.

1 One rheumatic fever, one labyrinthitis.

2 One varicella at 24 wks, one herpes zoster at 25 wks.

3 Tonsillitis. 4 Mumps. 5 Malaria. 6 Acute sinusitis.

TABLE VII.—*Infections in patients*, their sibs and control children*

| | ALL | | | | AML | | | |
|---|---|---|---|---|---|---|---|---|
| | Patients | Sibs | Patients + sibs | Control children | Patients | Sibs | Patients + sibs | Control children |
| No. | 54 | 84 | 138 | 121 | 28 | 38 | 66 | 67 |
| Infantile gastroenteritis | 4 | — | 4 | 2 | — | — | — | 1 |
| Pneumonia | — | 2 | 2 | — | — | — | — | — |
| Pyogenic infection | 4† | 2‡ | 6 | — | — | — | — | — |
| Upper respiratory infection | — | 1§ | 1 | — | 2 | — | 2 | 1 |
| Urinary infections | — | — | — | 3 | — | — | — | — |
| Infectious mononucleosis | — | 2 | 2 | — | — | — | — | 1 |
| Infective hepatitis | — | 1 | 1 | — | — | 1 | 1 | — |
| Viral meningitis | 1 | — | 1 | — | — | — | — | — |
| Tonsillectomy: No. | 3 | 16 | 19 | 10 | 2 | — | 2 | 5 |
| % | 5·5 | 19·0 | 13·7 | 8·5 | 7·0 | — | 3·0 | 7·5 |
| Total episodes of infection | 9 | 8 | 17 | 5 | 2 | 1 | 3 | 3 |
| Total children with infection | 6 | 8 | 14 | 5 | 2 | 1 | 3 | 2 |

* Before diagnosis of leukaemia.

† 2 patients had neonatal abscesses; one of these also had gastroenteritis and both developed abscesses at other sites at age 18 months and $3\frac{1}{2}$ years respectively.

‡ >10 boils each.

§ Operation for sinusitis.

or had a first-degree relative with, malignant disease, there were no statistically significant differences in any group (Tables III and IV). The types and sites of neoplasms were similar in patients' and control families, but chronic lymphocytic leukaemia occurred in 2 grandparents and AML in one grandparent of ALL patients, and in no relatives of controls.

*Blood tests*

No significant differences were found between paired counts of either circulating polymorphs, lymphocytes or monocytes, or for lymphocyte response to PHA in 22 ALL mothers, 22 ALL fathers, 7 AML mothers, 7 AML fathers, when compared to equal numbers of matched controls for each group.

No significant difference was found between parents and control parents for levels of serum IgG, IgA or IgM in any of the 3 samples from ALL parents and AML fathers. AML mothers showed significantly higher ($P<0{\cdot}02$ by paired $t$ test on logarithmically transformed values) IgM than controls in the first but not subsequent samples.

### DISCUSSION

The significantly ($P<0{\cdot}05$) increased number of reports of severe infantile infections in ALL parents and of diseases possibly associated with autoimmunity in ALL families ($P<0{\cdot}02$) support the concept of a primary immunological abnormality as an aetiological factor in ALL. Not only were autoimmune disorders reported significantly more often amongst relatives of ALL patients than amongst relatives of controls, but 5 members of patients' families suffered from more than one of these disorders and, in 8 compared with 2 control families, more than one individual per family was affected. Of the 108 parents of ALL patients 33% (36) had themselves had, or had a first-degree relative with, such a disorder compared with 18% of control parents. In contrast to these results, those for peptic ulceration and infective hepatitis (which are not considered to be of autoimmune origin and were included in the questionnaire in an attempt to control for reporting bias) and also the findings for atopy, diabetes, migraine, tuberculosis and malignant disease showed no statistical difference between ALL families and their controls.

The study was designed to exclude one-parent families, to simplify the procedure for selecting controls, but it is unlikely that this has seriously biased the results.

In only 3/7 families thus omitted was the exclusion due to the death of one parent, and it is perhaps of interest to note that one of these was of possible autoimmune origin, death being due to subacute bacterial endocarditis consequent upon previous rheumatic carditis.

No evidence for immunodeficiency in the parents of children with leukaemia was obtained from the blood-test results. Total and differential leucocyte counts, and lymphocyte transformation by PHA showed no significant differences between ALL and AML parents and their controls. These findings agree with those reported by Evans (1973) but do not confirm the finding of low monocyte counts in mothers and high basophil counts in fathers of ALL patients (Hann *et al.*, 1975). Serum IgM levels in AML mothers were significantly higher ($P < 0 \cdot 02$) than those of their controls for the first blood sample, but not in the later ones. The results in ALL mothers did not confirm the raised IgM reported by Sutton *et al.* (1969) and Chandra (1972), the raised IgG reported by Chandra (1972) and Hann *et al.* (1975) or the raised IgA reported by Hann *et al.* (1975). Evans (1973) similarly failed to find these differences.

Epidemiological data collected in an investigation of this kind must be interpreted with caution. The parents of children with leukaemia may recall illnesses in themselves and in their relatives more readily than the control parents who do not have the same incentive to do so. Some differences may thus occur between cases and controls which are not of aetiological relevance. The method of selecting the control families in this investigation might be considered unusual, but there is no reason to suppose that this introduced any specific reporting bias. The choice of friends of the index family is likely to have controlled quite well for social class, a factor which may influence the occurrence of some diseases. Also, because the controls were acquainted with the index child, their co-operation in completing the questionnaire is likely to have been more similar to that of the parents of the case, than would have been true of controls chosen in some other way. However, the controls may well have been selected by the patient's parents because of their willingness to help, and it is possible that this may have biased the findings in ways we cannot assess. Mothers of patients reported more infections during pregnancy than did control mothers, and more during their pregnancy with the affected child than in their other pregnancies. These differences may be partly due to biased recall but the confirmed reports of varicella and herpes zoster in 2 ALL mothers during their pregnancy with the patient are consistent with the experience of others. Stewart *et al.* (1958) found 2 cases of herpes zoster infection during 677 pregnancies involving children who subsequently developed leukaemia and one case in a similar number of control pregnancies. In other series, 2 of the children of 270 women who had had a varicella infection in pregnancy developed leukaemia, against 0·15 expected (Adelstein & Donovan, 1972), as did 3 children of 63 women similarly infected (Vianna & Polan, 1976). Taken together, these findings suggest that antenatal varicella may be an aetiological factor in some cases of childhood leukaemia.

The finding of more autoimmune disease in the families of leukaemia patients is of particular interest in view of the association between autoimmunity and leukaemia in NZB mice (Holmes & Burnet, 1963). This strain of mice is characterized by the presence of active Gross leukaemia virus in the tissues at birth and a rapid decline in T-lymphocyte function in the first few weeks of life (Ghaffar *et al.*, 1970). All these mice eventually succumb either to autoimmune disease or to leukaemia, and it has been postulated that both autoimmune disease and neoplasia are the result of hyperactivity of B cells in the absence of normal T-cell control (Gerschwin & Steinberg, 1973). The increased prevalence of autoimmune thyroid disorders (Aarskog, 1969)

and leukaemia (Stewart *et al.*, 1958; Holland *et al.*, 1962) in patients with Down's syndrome and of autoimmune thyroid disease amongst members of their families (Fialkow *et al.*, 1971) is suggestive of a similar situation in man, since those with Down's syndrome have been shown to have defective T-cell function (Agarwal *et al.*, 1970; Sutnik *et al.*, 1971). The increased prevalence of disorders associated with autoimmunity in relatives of ALL patients reported here suggests that genetic immunodeficiency may be a predisposing factor in childhood ALL. The lack of this finding in AML families remains unexplained, since the increased risk of leukaemia among patients with Down's syndrome is not confined to ALL, but applies to all cytological types (Lashof & Stewart, 1965). Further study of the cellular basis of the autoimmunity in ALL families will involve analysis of regulatory T-cell populations, particularly suppressor T cells.

We would like to thank Dr Judith Chessells for allowing us access to her patients and their families, all members of these families and the control families for their willing co-operation, Professor R. M. Hardisty and Professor J. F. Soothill for much advice and for criticism of the manuscript, Mr A. W. J. Bennett and Mr J. P. Okae for excellent technical help and Mrs Jean Bridger for typing the manuscript. The work was supported by grants from the Leukaemia Research Fund.

### REFERENCES

AARSKOG, D. (1969) Autoimmune thyroid disease in children with mongolism. *Arch. Dis. Child*, **44**, 454.

ADELSTEIN, A. M. & DONOVAN, J. W. (1972) Malignant disease in children whose mothers had chickenpox, mumps, or rubella in pregnancy. *Br. Med. J.* iv, 629.

AGARWAL, F. F., BLUMBERG, B. S., GERSTLEY, B. J. S., LONDON, W. T., SUTNIK, A. I. & LOEB, L. A. (1970) DNA polymerase activity as an index of lymphocyte stimulation: studies in Down's syndrome. *J. Clin. Invest.*, **49**, 161.

CHANDRA, R. K. (1972) Serum immunoglobulin levels in children with acute lymphoblastic leukaemia and their mothers and sibs. *Arch. Dis. Child*, **47**, 618.

EVANS, D. I. K. (1973) Immune response in families of children with acute lymphoblastic leukaemia. *Arch. Dis. Child*, **48**, 441.

FIALKOW, P. J., THULINE, H. C., HECHT, F. & BRYANT, J. (1971) Familial predisposition to thyroid disease in Down's syndrome: controlled immunoclinical studies. *Am. J. Hum. Genet.*, **23**, 67.

GERSCHWIN, M. E. & STEINBERG, A. D. (1973) Loss of suppressor function as a cause of lymphoid malignancy. *Lancet*, ii, 1174.

GHAFFAR, A., KRSIAKOVA, M. & PLAYFAIR, J. H. L. (1970) Deficient cell-mediated immunity in adult NZB mice. *Transplantation*, **10**, 432.

HANN, H. L., LONDON, W. T., SUTNICK, A. I. & 6 others (1975) Studies of parents of children with acute leukemia. *J. Natl Cancer Inst.*, **54**, 1299.

HOLLAND, W. W., DOLL, R. & CARTER, C. O. (1962) The mortality from leukaemia and other cancers among patients with Down's syndrome (mongols) and among their parents. *Br. J. Cancer*, **16**, 177.

HOLMES, M. C. & BURNET, F. M. (1963) The natural history of autoimmune disease in NZB mice: A comparison with the pattern of human autoimmune manifestations. *Ann. Int. Med.*, **59**, 265.

LASHOF, J. C. & STEWART, A. (1965) Oxford survey of childhood cancers progress report, III: Leukaemia and Down's syndrome. *Mth. Bull. Minist. Hlth.*, **24**, 136.

STEWART, A., WEBB, J. & HEWITT, D. (1958) A survey of childhood malignancies. *Br. Med. J.*, i, 1495.

SUTNICK, A. I., LONDON, W. T., BLUMBERG, B. S. & GERSTLEY B. J. S. (1971) Susceptibility to leukaemia: immunologic factors in Down's syndrome. *J. Natl Cancer Inst.* **47**, 923.

SUTTON R. N. P. BISHUN N. P. & SOOTHILL J. F. (1969) Immunological and chromosomal studies in first-degree relatives of children with acute lymphoblastic leukaemia. *Br. J. Haematol.*, **17**, 113.

TILL, M. M., JONES, L. H., PENTYCROSS, C. R., HARDISTY, R. M., LAWLER, S. D., HARVEY, B. A. M. & SOOTHILL, J. F. (1975) Leukaemia in children and their grandparents: studies of immune function in six families. *Br. J. Haematol.*, **29**, 575.

VIANNA, N. J. & POLAN, A. K. (1976) Childhood lymphatic leukemia: prenatal seasonality and possible association with congenital varicella. *Am. J. Epidemiol.*, **103**, 321.